# Meaningful coproduction with clinicians: establishing a practice-based research network with physiotherapists in regional Australia

**DOI:** 10.1186/s12961-023-00983-x

**Published:** 2023-05-26

**Authors:** Connor Gleadhill, Christopher M. Williams, Steven J. Kamper, Katarzyna Bolsewicz, Andrew Delbridge, Benjamin Mahon, Bruce Donald, Caitlin Delore, Craig Boettcher, David Renfrew, Joshua Manvell, Katherine Dooley, Michael Byrne, Toby Watson, Andrew Makaroff, Benedicta Gibbs, Christopher Barnett, Michael Corrigan, Murray Leyland, Nicholas Mullen, Ryan Gallagher, Samuel Zelinski, Steven Lamond, Travis Maude, Simon R. E. Davidson, Emma Robson, Priscilla Viana Da Silva, Nicole Manvell

**Affiliations:** 1 School of Medicine and Public Health, University of Newcastle, Newcastle, Australia; 2 Hunter New England Population Health, Hunter New England Local Health District, Newcastle, Australia; 3 School of Health Sciences, University Centre for Rural Health, University of Sydney, Camperdown, Australia; 4 Mid North Coast Local Health District, Port Macquarie, Australia; 5 School of Health Sciences, University of Sydney, Camperdown, Australia; 6 Nepean Blue Mountains Local Health District, Nepean Hospital, Penrith, Australia; 7 National Centre for Immunisation Research and Surveillance, Kids Research, Sydney Children’s Hospitals Network, Westmead, Australia; 8 Regent Street Physiotherapy, New Lambton Heights, Australia; 9 Unified Health, Newcastle, Australia; 10 John Hunter Hospital Physiotherapy, Hunter New England Local Health District, New Lambton Heights, Australia; 11 Newcastle Performance Physiotherapy, Newcastle West, Australia; 12 Department of Orthopaedic Surgery, John Hunter Hospital, Hunter New England Local Health District, New Lambton Heights, Australia; 13 School of Allied Health, Exercise and Sport, Charles Sturt University, Bathurst, Australia; 14 Recovery Partners, Newcastle, Australia; 15 The Good Physio, Canberra, Australia; 16 Employers Mutual Limited, Sydney, Australia; 17 APM Workcare, Auckland, New Zealand; 18 ORS Group, Newcastle, Australia; 19 Atune Health Centres, Newcastle, Australia; 20 Thornton Physiotherapy, Maitland, Australia; 21 Ethos Health, Newcastle, Australia; 22 NuMoves Physiotherapy, Newcastle, Australia; 23 NextGen Physiotherapy, Newcastle, Australia; 24 Advanced Physiotherapy, Newcastle, Australia

**Keywords:** Coproduction, Collaborative research, Research impact, Physiotherapy, Evidence-based practice

## Abstract

**Background:**

The disconnect between research and clinical practice leads to research evidence that is often not useful for clinical practice. Practice-based research networks are collaborations between researchers and clinicians aimed at coproducing more useful research. Such networks are rare in the physiotherapy field. We aimed to describe (i) clinicians’ motivations behind, and enablers to, participating in a network, (ii) the process of network establishment and (iii) research priorities for a practice-based network of physiotherapists in the Hunter Region of New South Wales (NSW), Australia that supports research coproduction.

**Methods:**

We describe the methods and outcomes of the three steps we used to establish the network. Step 1 involved consultation with local opinion leaders and a formative evaluation to understand clinicians’ motivations behind, and enablers to, participating in a network. Step 2 involved establishment activities to generate a founding membership group and codesign a governance model. Step 3 involved mapping clinical problems through a workshop guided by systems thinking theory with local stakeholders and prioritizing research areas.

**Results:**

Through formative evaluation focus groups, we generated five key motivating themes and three key enablers for physiotherapists’ involvement in the network. Establishment activities led to a founding membership group (*n* = 29, 67% from private practice clinics), a network vision and mission statement, and a joint governance group (9/13 [70%] are private practice clinicians). Our problem-mapping and prioritization process led to three clinically relevant priority research areas with the potential for significant change in practice and patient outcomes.

**Conclusions:**

Clinicians are motivated to break down traditional siloed research generation and collaborate with researchers to solve a wide array of issues with the delivery of care. Practice-based research networks have promise for both researchers and clinicians in the common goal of improving patient outcomes.

**Supplementary Information:**

The online version contains supplementary material available at 10.1186/s12961-023-00983-x.

## Background

The disconnect between research and clinical practice leads to a substantial amount of research that fails to improve practice or patient outcomes [1–3]. Typically, researchers set research agendas and design research questions in isolation. The failure to involve end-users in setting research agendas and designing research questions has resulted in proliferation of research that clinicians do not find relevant [4–7]. Translating findings into practice is also more difficult when researchers and clinicians work in silos [1–3].

Involving end-users (e.g., patients, community and clinicians) in research is one way to improve research translation [8–12]. End-user involvement, often called coproduction, refers to a variety of practices where researchers work with stakeholders to generate research evidence. Mounting evidence suggests that involving end-users in producing research improves its relevance and likelihood of implementation into practice [8–12]. Moreover, there is a strong ethical rationale for involving end-users with the ability to shape the research that is designed to assist them [13, 14]. However, end-user involvement may require more effort than traditional research processes [15–17]. Power imbalances and structural barriers concerning how research is typically conducted can lead to tokenistic collaborative efforts and involvement arrangements [18, 19].

Practice-based research networks are ongoing collaborations between researchers and clinicians that aim to support coproduction [20]. In practice-based research networks, clinicians are active in setting research agendas and conducting research [20, 21]. Practice-based research networks have been proposed to conduct more relevant research, accelerate research findings into practice and connect clinicians to key care stakeholders such as policy-makers and funders of care [20, 22, 23]. Practice-based research networks are common in general practice [21, 24], but there are only a few select examples of practice-based research networks for physiotherapists in Australia [25, 26]. There is a lack of information available to guide clinicians or researchers in establishing their own practice-based research networks.

To address this lack of information, we aimed to describe (i) clinicians’ motivations behind, and enablers to, participating in a network; (ii) the process of network establishment and (iii) research priorities for a practice-based network of physiotherapists in the Hunter Region of New South Wales (NSW), Australia, that supports research coproduction.

## Methods

### Design

**Fig. 1:**
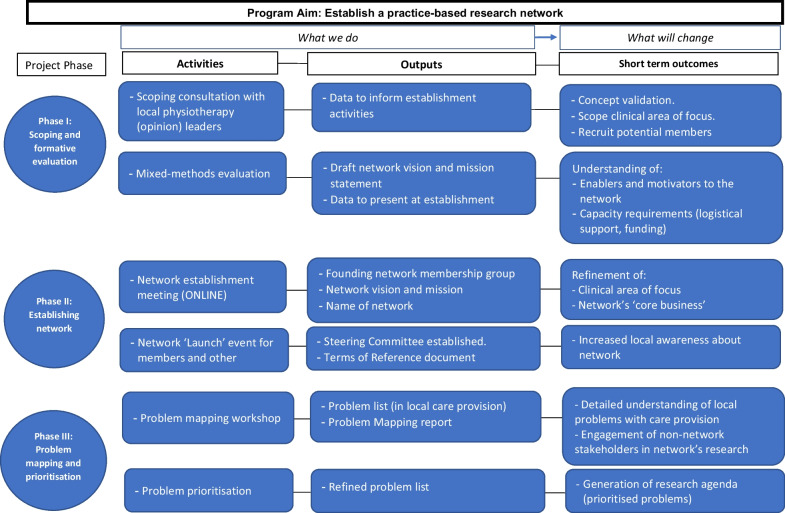
Program logic guiding the network establishment. The top row lists the logic guiding the activities and outputs and theorized outcome and impacts for the overall program [27]. *Formative evaluation resulted in data used in two research reports. Motivators of the network and reported enablers to a successful network are reported here, and barriers and enablers to providing evidence-based care are reported elsewhere [29].

We used program logic to design key activities and identify outputs in a three-step process (Fig. 1) [27]. First, we performed a qualitative study as part of a formative evaluation to assess problems with care provision and ways that a research network could address these problems (we refer to these as motivators). We also assessed enablers for participation in a practice-based research network. Second, we carried out two activities to develop and formally establish the network and used codesign to develop network governance [28]. Finally, we performed problem mapping through a face-to-face workshop and prioritization of research areas through an online survey.

### Coproduction and codesign

Increasing enthusiasm for partnering with end-users has led to conflation and misappropriation of “co-” terms such as coproduction and codesign [13, 18]. For the purposes of our study, we defined coproduction as researchers and clinicians collaboratively generating research [11–14]. We defined codesign as a method where we worked with clinicians in multiple steps to create an end-product [28, 29]. We accept that involving patients in coproduction and codesign is key to success [8–13, 18]. However, the focus of this paper was to establish a network for clinicians, and our use of the terms coproduction and codesign throughout this manuscript refers to involving only researchers and clinicians.

### Ethical considerations

The entire study was approved by the Hunter New England Local Health District Human Research Ethics Committee (Reference number: 2020/ETH01029).

### Step 1: scoping and formative evaluation

We performed an initial scoping consultation with local physiotherapy opinion leaders prior to formative evaluation, which involved an online survey and online focus groups. Online focus groups are reported here and represent one portion of the formative evaluation. More detail on formative evaluation is reported elsewhere [30]. Here, we only report focus group data that address physiotherapists’ thoughts and perceptions of a network and its establishment. The group interaction of focus groups was deemed the ideal way for participants to share ideas about their problems with care delivery and thoughts about how the network might address these problems, and to propose ideas about how the network might function [29, 31].

#### Participants

Initial scoping was targeted to local clinical opinion leaders who were defined as either physiotherapists with extensive clinical experience (over 15 years) or people with a high degree of centrality in professional networks and who were capable of being “change agents”. [32, 33]

To be eligible for the focus groups, participants had to be registered physiotherapists working in regional or rural NSW [34]. We purposely sampled physiotherapists from three settings: private practice, public health system and physiotherapist researchers. We invited participants via individual emails, which included information sheets about the study (Additional file 3: Supplement 1).

#### Activities

Initial scoping: We consulted local physiotherapy opinion leaders to assist in the design of subsequent stages [32], validate the network concept and understand the general scope of the network.

*Focus groups:* The facilitation schedule (Additional file 1) explored four main questions: (1) How do we improve practice? (2) Where does research fit into improving practice? (3) What are the issues that this network could help with? (4) How do you see this network being successful?

We conducted four focus groups online using Zoom [35] between 29 July and 6 August 2020. Each focus group lasted approximately 60 minutes. Two researchers facilitated each group, composed of four to six physiotherapists (a maximum of eight participants in each focus group).

One researcher (CG) transcribed audio files verbatim from each focus group, cleaned transcripts, and de-identified and organized data in NVivo software [36]. CG descriptively coded focus group data, and codes were checked by a second author (SD) [37, 38]. CG led the development of themes with inductive thematic analysis [38, 39]. During analysis, CG grouped codes under “problems” and “solutions” and explored the relationships between them to develop overarching categories. Motivating themes, or “motivators”, resulted from grouping similar categories of problems and solutions together. “Enablers” were developed by grouping together common categories of participant-reported ideas on what would make their participation in the network easier [37–39]. Themes were finalized through discussion among CG, KB, SK and CW.

CG is a physiotherapist with lived experience of the challenges that were reported by participants. CG has professional relationships with many focus group participants. These factors have shaped data collection and analysis, and theme development.

### Step 2: establishment activities

First, we developed a clinician-led vision and mission statement and formed a founding membership group through an online network establishment meeting. Second, we codesigned a joint governance group and held a launch event to raise local awareness of the group. We used codesign because it allows multiple iterations to create products in partnership with end-users, where the outcome of the design process better meets end-users’ needs and respects their experience [16].

#### Participants

We determined eligibility for the network as being a registered physiotherapist or a professional who contributes to physiotherapy care (for example, a rehabilitation provider), and working in the greater Newcastle, Lake Macquarie and lower Hunter regions of NSW. This sample was selected to ensure that network members could meet and interact regularly with shared interests in improving local patient outcomes. Recruitment efforts can be described as convenience sampling. We focused recruitment efforts on a list of potential members that emerged from initial scoping (more information under results section). We primarily used face-to-face meetings, online meetings, email or phone calls to recruit participants. Supplementary recruitment strategies included social media posts, social media advertisements (targeted to regional physiotherapists in NSW), and a website landing page to advertise the network. We invited 45 potential members to both establishment activities.

#### Activities

Network establishment meeting: Prior to the meeting, CG thematically analysed data from step 1 to generate a draft vision and mission statement, which was provided as pre-reading for participants. We determined consensus for the vision and mission statement as all participants present were in agreement (100% agreement).

We invited potential members to an online meeting (6 November 2020). After receiving background information on practice-based research networks, participants discussed the vision and mission statement. Participants who disagreed suggested changes, and the groups discussed these changes until we reached 100% agreement. We provided participants with an opportunity to make small, grammatical edits to the vision and mission statement after the meeting through a live online document.

We then asked participants to consider being a founding member of the network and encouraged them to confirm their response through an online communications platform (Slack) [40]. Following the establishment meeting, we took suggestions for the network name and performed an online poll (through Slack) [40].

*Launch event:* Prior to the launch event, a small group of founding members (CG, AD, NM, BG, KD, CW) discussed different leadership frameworks over three successive meetings and made a shared decision on the final model. We invited network members and local care stakeholders to a launch event (3 December 2020) to create awareness about the network and gather expressions of interest to become part of the network’s steering committee. Following the launch event, the steering committee held sequential meetings to design terms of reference and make key decisions about the network scope.

### Step 3: problem mapping and prioritization

We first held a face-to-face workshop to list and explore the causes and effects of key problems with the delivery of care for patients with musculoskeletal conditions in the local community. We used systems thinking to design the workshop [41]. The network steering committee then prioritized problems that resulted from the workshop.

#### Participants

We wanted to gain perspectives from professionals who provide musculoskeletal care in different parts of the local health system [41]. Therefore, we invited network members plus local emergency department consultants and nurse practitioners, general practitioners, orthopaedic surgeons and sports physicians to the problem-mapping workshop. Network members and the steering committee were involved in the prioritization process.

#### Activities

*Workshop:* Patients with musculoskeletal conditions are managed within a health system composed of multiple interacting agents (different professionals in different health sectors). Hence, we based our workshop on general systems theory, which broadly deals with exploring the role of interacting agents and their connections [37]. During the workshop we encouraged participants to consider links between parts of the system, and how these links (or lack of) influence major problems. Prior to the workshop, all participants were asked about their perspectives on the main problems they face with care delivery for patients with musculoskeletal conditions and the criteria they would consider for setting priorities. CG analysed these responses and created a list of key problem areas and criteria for prioritizing problems by gathering similar responses under common categories. Criteria for prioritizing problem areas were used as a rough guide for participants to consider when making their decisions. In the first half of the workshop, participants reviewed and added to the problem list from the pre-workshop survey, then prioritized six key preliminary problem areas. In the second half of the workshop, participants split into groups (one group per key problem area) and discussed the causes, effects, what is known or unknown, and potential strategies to address the problem area. We captured responses on paper.

*Prioritization:* Workshop data and field notes were analysed to produce a report for the workshop participants. Network members were asked to reflect on this report and provide their feedback through a live online document. For pragmatic reasons (we wanted to demonstrate meaningful progress on generating research with our limited capacity), we chose to prioritize the final three problem areas through an online poll involving only steering committee members. Steering committee members were asked to vote for three areas, without ranking them. We did not ask steering committee members to consider any specific criteria when ranking problem areas.

## Results

### Step 1: scoping and formative evaluation

#### Participants

Sixteen people were involved in the focus groups (Additional file 2). Focus group participants were on average 39 (30–48) years of age, with a range of clinical experience [1–5 years of experience—2/16 (12%); 6–10 years—5/16 (31%); 11–15 years—3/16 (20%); 16–20 years—2/16 (12%); 21+ years—4/16 (25%)], were mostly practicing in a regional location [14/16 (88%)], and had a musculoskeletal focus [11/16 (69%)].

Initial scoping led to a list of potential network members who were already part of a professional network with a large number of pre-existing connections or who had professional relationships with local opinion leaders. We chose this list of potential members to optimize the diffusion of information and innovation [34].

### Findings

#### Motivators to becoming involved in the network

We report each theme as “motivators”, as solutions that mapped to problems with evidence-based care delivery reported by physiotherapists (except in the case of theme 5—making local impact) (Table 1).

**Table 1:** Problems with providing evidence-based care, and potential solutions (motivators for becoming involved in the network)

Problems with providing evidence-based care	Solutions that could be enabled by the network (motivators)
*Research is not relevant* • Research that asks the wrong questions • Research that doesn’t capture “real-world” complexity	*Improve research relevance* • Asking clinically relevant questions • Making treatments included in research more implementable in the “real world”
*Disconnected systems* • Research practice divide • Limited interprofessional communication • Funders are not connected to clinicians	*Enabling connection and collaboration* • Connect clinicians with researchers, other professionals, funders and other stakeholders • Collaborate with each other rather than compete
*Care variability* • Physiotherapists all do things differently • There is no system to keep clinicians accountable to a particular standard of care	*Improving accountability* • Creating a culture of excellence • Establishing a system of accountability
*It is hard to market evidence-based care* • It is hard to promote the difference between evidence-based and non-evidence-based care	*Promoting evidence-based care* • A practice-based research network is a marker of quality for physiotherapists, which can be promoted
	*Making local impact* • Improving the care for local patients • Promoting their regional healthcare community

#### Theme 1: improving research relevance

Most participants reported that researchers may be asking the wrong questions to guide clinical practice improvement. Participants noted that the network may be a way for researchers to make research questions more relevant to clinical practice. For example, one participant reported,

*I sort of need researchers to better understand what clinic life is like. So that they’re asking better questions.* (Participant 16, focus group 4)

Some participants noted that treatments included in research may be difficult to implement in “real-world” settings because protocols are too rigid, which does not reflect the realities of clinical practice. Participants reported that the network may improve the implementability of research. For example, one participant reported,

*It [network research] needs to be scientific obviously for it to be a high-quality paper. But make it so that it’s able to be implemented and followed by people.* (Participant 6, focus group 2)

#### Theme 2: enabling connection and collaboration

Many participants shared that disconnected systems are a hindrance to care provision. Disconnections between research and practice, between care providers, and between funders of healthcare and clinicians were reported. Participants reported that a network may improve the chances of clinicians connecting with healthcare funders, by providing a united voice (in contrast to separate individuals approaching healthcare funders).

*I think, collectively, if we had something that we could—you know—as a collective go, well, this is what we’re doing and that would then allow us to have a bit of a seat at the table when those conversations are happening [with funders of healthcare].* (Participant 15, focus group 4)

Often participants noted that providing care in a market-driven primary care environment forces physiotherapists to compete for patients’ business. However, participants reported that the network could enable collaboration among clinicians, by sharing information with the shared goal of improving patient outcomes.

*If we’re really looking at maximizing the outcomes for our patients but yet still sort of remaining happy, healthy physiotherapists, we’ve got to make sure that information about what works and what doesn’t is shared across the profession as much as possible.* (Participant 7, focus group 3)

#### Theme 3: improving accountability

Some participants reported that a problem in physiotherapy is the variability in care that a patient will receive between physiotherapists because of a lack of obligatory standards. Participants discussed that the network could reduce unnecessary care variability in their local region by enabling the measurement of care standards and establishing a culture of accountability.

*We [physiotherapists] are first-contact practitioners, so we do have that responsibility, and yet we don’t necessarily, whether it’s public or private, have the infrastructure and maybe the really deep-seated culture of accountability…But out of this network we are able to have some of those self-imposed accountability structures.* (Participant 10, focus group 3)

#### Theme 4: promoting evidence-based care

Most participants reported that evidence-based care is difficult to market, because patients may not understand the difference between care that is underpinned by evidence and other care options with less empirical support. Participants noted that being a part of the network would be a marker of quality and a unique selling proposition for their services that are underpinned by evidence. Participants shared that their (evidence-based) services are currently indistinguishable from service providers that do not practice in an evidence-based manner. A network “affiliation, branding or stamp” may assist in competing for business and promoting their services to patients. For example, a participant shared that promoting evidence-based care can reflect well on the physiotherapy profession more broadly.

*People are seeing that there is this high degree of quality coming out constantly and we say it’s physio-driven.* (Participant 14, focus group 4).

#### Theme 5: making local impact

Some participants shared that the desire to have an impact on the care of patients in their local region was important, and that the network could be a vehicle to achieve this impact.

*Trying to make a difference in Newcastle and the care of patients with musculoskeletal, sporting problems.* (Participant 3, focus group 1)

Participants also reported that promoting themselves and the local healthcare community through involvement in research was important.

*I think having some sort of local, Newcastle-based network-based thing, there’s something nice about that. Trying to promote physio within the Newcastle region. Me being involved in that would be pretty cool.* (Participant 6, focus group 3)

#### Enablers to a practice-based research network

#### Theme 1: “time-crunched”

Participants reported that ensuring that network activities fit around a busy clinic schedule (“time-crunched”) would make it easier for them to partake in network activities. One participant shared,

*I think if a clinician is really interested, they have to be interested in engaging with research, then they have to actually take time out of their clinical schedule to do that.* (Participant 9, focus group 3)

#### Theme 2: research infrastructure support

Participants noted that having infrastructure to support research activities would enable their involvement. Participants shared that staffing support to undertake research activities and funding support to cover the costs of research would be important enablers. For example, one participant shared,

*Where’s the money gonna come from? It’s not just money, is it? It’s resources and time.* (Participant 4, focus group 2)

#### Theme 3: motivation and commitment

Participants noted that those who are more motivated to engage in research are more likely to be involved. However, participants also reported that clinicians who are committed, as well as motivated, may make the network a success. One participant noted,

*We need some really motivated individuals.* (Participant 5, focus group 2)

And another shared,

*And a commitment, I suppose, as well. Behaviour change amongst clinicians is as important as patients.* (Participant 3, focus group 1)

### Step 2: network establishment

Details of the outputs from network establishment activities are provided in Table 2.

**Table 2:** Establishment activity results

Activity	Output
Network establishment meeting	Founding membership (*n* = 29)^a^: Health sector Private practice (20/29 [69%]) Public health (3/29 [10%]) Private health insurance or industry professionals (4/29 [14%]) Research (2/29 [7%]) Professional group Physiotherapists (27/29 [93%]) Medical officers (1/29 [3.5%]) Other professionals (1/29 [3.5%]) Vision and mission statements (100% agreement) *Our vision:* To improve the care that WE DELIVER and create lasting improvements in patient outcomes in our local community. [Changed from *Our vision is to improve the care that our patients receive by collaborating to create lasting change in our local healthcare community*] *Our mission:* We aim to: • Connect and collaborate to promote change in our healthcare community • Generate clinically relevant research that makes meaningful differences in our patients’ lives • Break down barriers between research and clinical practice physiotherapy, across disciplines and between stakeholders (like consumers and care funders) [No changes made by participants] Network name: Research in Practice Network
Launch event	Steering committee membership (*n* = 13) Practising physiotherapists from private practice (9/13 [70%]) Researchers (2/13 [15%]) Medical officers (1/13 [7.5%]) Industry professionals (rehabilitation provider) (1/13 [7.5%])

^a^CG, CW, AD, BM, BD, CD, CBo, DR, JM, KD, MB, TW, AM, BG, CBa, MC, ML, RG, SZ, SL, TM, NM are network founding members. Seven other founding members are not listed as coauthors (they did not meet the International Committee of Medical Journal Editors [42] requirements to be listed as coauthors)

#### Members

There were 29 founding members of the network, which was 64% of potential members. Twenty-seven (93%) of the founding members are registered physiotherapists (the two founding members who are not physiotherapists include one rehabilitation provider and one medical doctor). Twenty (69%) members worked in private practice, representing 16 private practice clinics in Newcastle, Lake Macquarie and Maitland, NSW. Five members worked in senior clinical and managerial roles at the local health district (Hunter New England Local Health District). One member worked in a private health insurance company, and one worked for a rehabilitation provider. Three researchers were founding members of the network (CG, CW, KD).

### Findings

*Network establishment meeting:* The network’s vision and mission statement reached consensus (100% agreement of meeting participants) after key changes were made to emphasize the desire for network members to improve their own care quality (changing “the care that patients receive” to “the care that WE provide”). We established a founding network membership group and finalized the network name 1 week after the meeting.

*Launch event:* The final leadership model we designed was a steering committee consisting of clinicians, researchers and other care stakeholders capable of providing a unique perspective on the healthcare system. Following the launch event, we received 14 expressions of interest; 13 of these became steering committee members (AD, BD, BM, CD, CG, CW, DR, JM, KD, MB, NM, SL, TW). The steering committee has majority (9/13 [70%]) representation from physiotherapists who work full-time in private clinics, and minority (2/13 [15%]) representation from researchers (Table 2). To simplify the research agenda, the steering committee initially limited membership to physiotherapists, with a view to incorporating other disciplines in the coming years. The steering committee limited the network’s scope to musculoskeletal conditions because the network membership predominantly practised in the musculoskeletal area of practice.

### Step 3: problem mapping and prioritization

See Table 3 for responses from the first half of the workshop (listing problems and preliminary prioritization plus the criteria provided in the pre-workshop survey). The following three areas were prioritized by the network steering committee through an online poll to contend with clinicians’ busy schedules:

(i) public and patient perception of musculoskeletal conditions and what is effective to manage them,
(ii) poor quality of care that patients with musculoskeletal conditions receive,
(iii) lack of preventative focus from the health system.

**Table 3:** Problem-mapping workshop problem areas and criteria for prioritization

Preliminary priority problem areas^a^	Criteria used to prioritize problem^a^	Final research priorities^b^
(1) Public and patient perception of musculoskeletal conditions and what is effective to manage them (2) Poor quality of care that patients with musculoskeletal conditions receive (3) Lack of preventive focus in the health system (4) Issues with funding model (5) Patient compliance to care (6) Resource availability	• Impact on the patient (4/8 responses [50%]) • Ease of tackling/“low-hanging fruit” (4/8 responses [50%]) • Impact on therapist (2/8 responses [25%]) • Frequency of problem occurring [1/8 responses (12.5%)] • Burden of the problem on the health system [1/8 responses (12.5%)]	(1) Public and patient perception of musculoskeletal conditions and what is effective to manage them (2) Poor quality of care that patients with musculoskeletal conditions receive (3) Lack of preventive focus in the health system

^a^Criteria were used only to guide the decision-making for participants (participants did not score problem areas using these criteria). ^b^The six preliminary problem areas were then prioritized by the network steering committee via online poll

### Future research directions

A foundational research program has been developed based on the priority areas identified (Additional file 3). Initial projects completed include a rapid review and a consensus project [46]. Our research areas are intentionally broad, and we recognize that specific and answerable research questions are needed to undertake research with tangible clinical impact and translation potential (e.g. research-embedded clinical or implementation trials). Our process to refine project areas includes assessing the literature base for knowledge gaps and specifying a clear research question in an iterative process. To coproduce research, we used small working groups including experienced researchers and clinicians, with one-to-one mentoring to build clinicians’ research capabilities (for example, performing a literature search on a scientific database).

The network faces several ongoing challenges to support and sustain activity. Maintaining network member engagement and securing adequate resources and funding for research are two ongoing challenges. To boost member engagement, we have established a clinically relevant professional development program that includes research capacity-building. For funding research, we have developed a detailed funding strategy. The strategy outlines our approach to identifying and applying for relevant grant opportunities, partnership options, the development of a track record to secure independent funding, and alternative forms of income (e.g. professional development events, memberships).

## Discussion

Our formative evaluation showed that physiotherapists were motivated to contribute to a research network to coproduce research that is more relevant to clinical practice and to improving patient outcomes more broadly. We achieved involvement from 29 founding members across 16 practices, who codesigned governance structures, mapped systemwide problems with care delivery priorities and generated research priorities. The network was viewed as a vehicle to connect disconnected health systems through knowledge sharing, promote evidence-based care against other non-evidence-based alternatives, improve the overall standard of care delivered by physiotherapists and have an impact in the local community. Physiotherapists reported that “time-crunched” network activities, research infrastructure support, and motivated and committed members would enable a successful network. Establishment activities led to key outputs such as a vision and mission statement to harness physiotherapists’ motivations and a joint governance group. These data may help other researchers or clinicians who wish to form a practice-based research network and coproduce research.

### Interpretation

Physiotherapists’ motivations extended beyond generating research, suggesting that a network is viewed as a way to address an array of problems with care delivery. In our study, all motivating factors shared one common theme: the underlying link to improving patient outcomes. Evidence from similar initiatives aimed at connecting clinicians and researchers demonstrates a similar patient-centred motivation [43]. Ultimately, the integration of high-quality research and clinical experience—traditionally outlined in evidence-based practice—is a cornerstone of healthcare that aims to optimize patient outcomes [44]. Working together through a mechanism such as a practice-based research network may create additional value for researchers and clinicians in the common pursuit of better evidence-based practice and improving patient outcomes.

### Practical takeaways

To achieve a clinically relevant research agenda, we used a joint governance model with majority representation from private practice physiotherapists [9/13 (70%)]. To allow maximal engagement from busy clinicians, we balanced pragmatism with rigour in establishment activities. For example, we used a brief online poll to prioritize research areas rather than following a Delphi process [45]. Activities were typically scheduled in the evening (after clinical hours) and were less than 2 hours in length. We used a mix of online and face-to-face meetings based on the preferences of network members, and we codesigned aspects of the network through online documents to ensure time efficiency.

### Strengths

Our study involved a stepped approach using principles of codesign. While iterative in nature, our transparent description allows others to replicate or adapt specific components to their settings. We are not aware of another study describing the approach and results of research network establishment. For our formative evaluation, we purposively sampled physiotherapists from a variety of backgrounds, which should improve the transferability of our findings. Our manuscript was written in partnership with network members, which ensures an accurate reflection of included clinicians’ perspectives and the establishment process.

### Limitations

For our formative evaluation, CG has experience with the motivators we present and had professional relationships with participants, which may impact the credibility and confirmability of our findings. However, codes were checked by a second author (SD) and themes were discussed among a group of authors. We have only presented motivations for physiotherapists to become involved in the network, and not researchers’ motivations (which would presumably be quite different from clinicians’ motivations). However, we assume that collaboration with clinicians is understood by researchers as advantageous for many normative and functional reasons. For the network establishment, some activities may be difficult to apply to other contexts without the pre-existing social infrastructure or support personnel to implement establishment activities. However, our transparent description was intended to serve as a framework for others to adapt to their context, not a recipe to be followed. Finally, patients have an integral role to play in coproducing research, and a limitation in our study is that we did not include them in formative evaluation or in network establishment activities. However, the network is already involving patient as research partners to ensure a meaningful partnership moving forward.

## Conclusions

Physiotherapists’ motivations to be involved in a practice-based research network extended beyond generating research and centred around improving patient outcomes more broadly. Collaboration in a network such as this may allow clinicians and researchers to identify and address a wider array of issues with care provision than is possible through traditional practices. This suggests that practice-based research networks may have added value for both clinicians and researchers towards the common goal of improving patient outcomes. Further work should explore the proposed benefits of a practice-based research network and understand how such networks can be adapted to other contexts.

## Supplementary Information

**Additional file 1.** Focus group information sheet and interview schedule.

**Additional file 2.** Focus group participants.

**Additional file 3.** Overview of the Network's initialresearch program.

## Data Availability

Deidentified participant data are available from corresponding author upon reasonable request.
