# Supplementary material for: Meaningful coproduction with clinicians: establishing a practice-based research network with physiotherapists in regional Australia
Source: Health Res Policy Syst. 2023 May 26;21:38. doi: 10.1186/s12961-023-00983-x (PMC10223842; doi:10.1186/s12961-023-00983-x)
Supplement: Supplementary file 1 — Additional file 1. Focus group information sheet and interview schedule. [file 12961_2023_983_MOESM1_ESM.docx]

# Additional file 2

| *Focus groups participants (n=16)* | |
| --- | --- |
| **Age (years, SD)** | 39 (30-48) |
| **Years in practice**  1-5  6-10  11-15  16-20  21+ | 2 (12%)  5 (31%)  3 (20%)  2 (12%)  4 (25%) |
| **Location of practice**  Regional  Rural  Remote  Don’t know | 14 (88%)  1 (6%)  0 (0%)  1 (6%) |
| **Clinical area of practice**  Cardiothoracics  Chronic pain  Chronic respiratory disease  Continence and Women’s Health  Gerontology  Musculoskeletal  Neurology  Oncology  Orthopaedics  Sports  Whiplash | 0 (0%)  1 (6%)  0 (0%)  0 (0%)  0 (0%)  11 (69%)  1 (6%)  0 (0%)  0 (0%)  3 (19%)  0 (0%) |
