# Supplementary material for: Meaningful coproduction with clinicians: establishing a practice-based research network with physiotherapists in regional Australia
Source: Health Res Policy Syst. 2023 May 26;21:38. doi: 10.1186/s12961-023-00983-x (PMC10223842; doi:10.1186/s12961-023-00983-x)
Supplement: Supplementary file 2 — Additional file 2. Focus group participants. [file 12961_2023_983_MOESM2_ESM.docx]

**Additional file 3:** Overview of the Network’s initial research program


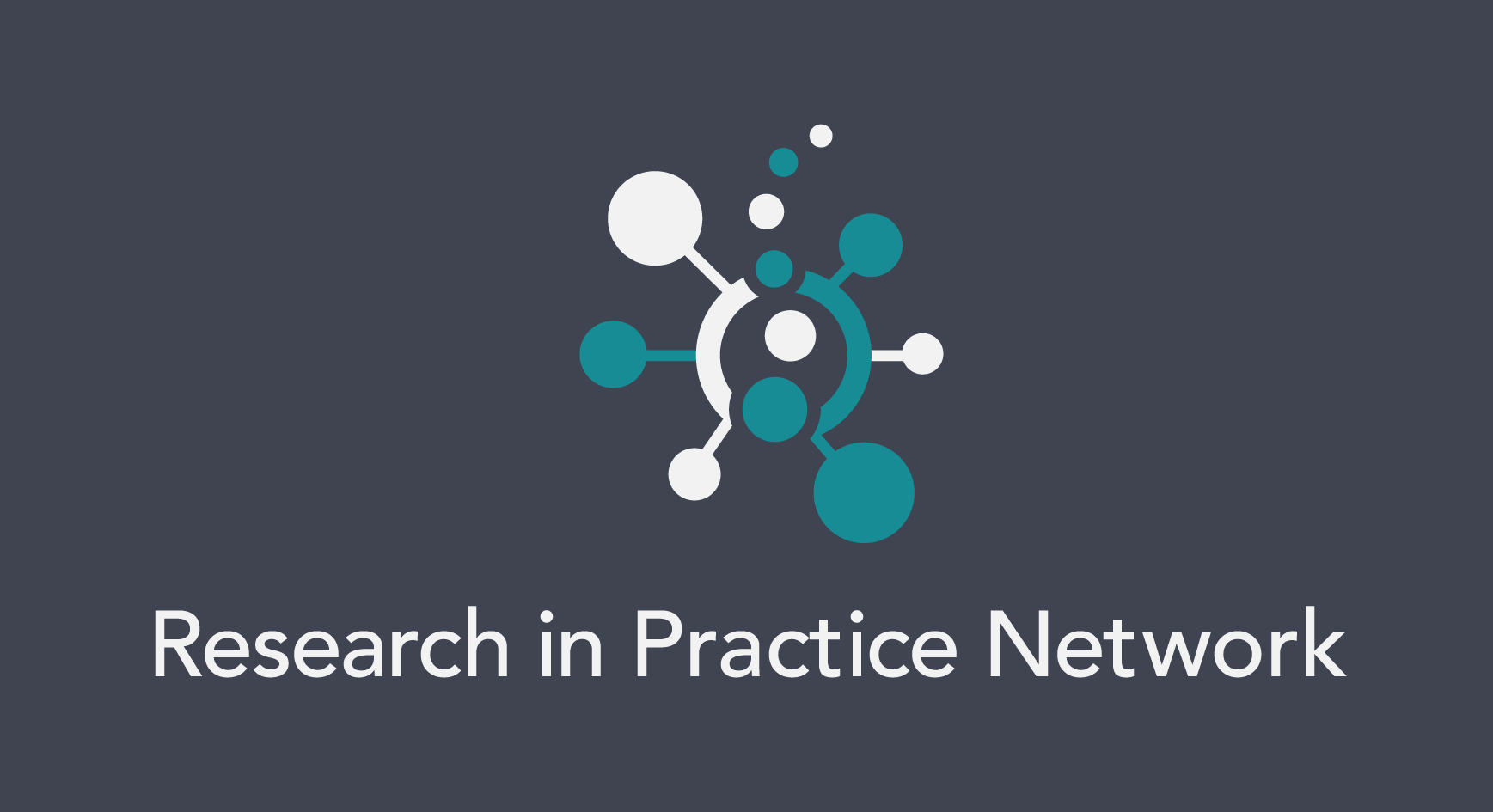
**Research Program**

This program has emerged from problem mapping by Research In Practice Network (RIPN) members and subsequent problem area prioritisation by RIPN Steering Committee.

**Public and patients’ perception of musculoskeletal conditions and what is effective to manage them**

**Poor quality of care for musculoskeletal conditions**

**Lack of preventive focus from healthcare system**

3 ‘Pillars’: Public and patient involvement, quality of care, prevention

**RIPN Research Program**

***Members of the Research Proposal Working Group:*** Connor Gleadhill, Bruce Donald, Chris Williams, David Renfrew, Katherine Dooley, Michael Byrne, Priscilla Da Silva, Simon Davidson.

**Executive Summary**

***The problem/s****:* Musculoskeletal (MSK) conditions have remained the leading cause of disability around the world for over two decades. Within the footprint of RIPN - regional New South Wales (NSW) - MSK conditions continue to cause significant burden for the local community. Despite emerging knowledge of the best way to manage MSK conditions, care in regional NSW remains variable. High value care and high-quality care are two terms that encompass ‘standards’, to which practitioners strive for when treating patients with MSK conditions. Theoretically, every patient should receive high quality and high value care, however, there are difficulties in ensuring this happens in practice.

***What we know:*** High quality and high value care are often used as synonyms for guideline recommended care or ‘evidence-based practice’. Although linked, the concepts of high-quality care, high value care and ‘evidence-based practice’ are not the same. High value and high-quality care are characterised by incorporating patient needs and values, and ‘evidence-based practice’ is the judicious use of evidence to inform practice. Clinical practice guidelines are one form of evidence, which evidence-based practitioners can use to inform their practice. While guidelines make recommendations about care based on the best available evidence, they do not consider the appropriateness, generalisability and challenges of delivering care in the local context for individual patient needs and values. Clarity on the terms, overlap and differences between high quality, high value care and evidence-based practice is a prerequisite to enable clinicians to achieve these ideals. Then it is key to understand how individual patient needs and values can be incorporated in the local context. Anecdotally, patients seem to have preferences for what health providers and funders might consider low value or ineffective care. Understanding more about patient’s preferences is integral to moving forward together.

***What we don’t know:*** To improve the provision of high-quality care or high-value care, we first need to understand more about the context of care delivery. This might include:

What do physiotherapists, patients and other clinicians consider to be the elements of high value and high-quality care?

What are the challenges to delivering the elements of high quality and value-based care?

What do physiotherapists, patients, and other clinicians consider the elements of low value or harmful care?

What does patient-centredness mean in physiotherapy, and how do they do it?

What is prevention in usual physiotherapy practice, and how do they do it?

What strategies effectively influence improvement in quality or value in the provision of care (for physiotherapy practice or care for musculoskeletal conditions)?

***Why is the work important****:* Through this work, we will gain clarity on the concepts being discussed for RIPN research (three ‘pillars’; quality of care, public and patient involvement and prevention). The approach will help identify targets for strategies used to influence the delivery of care for MSK conditions. The long-term vision for RIPN would be to assess the effectiveness of strategies and in turn influence the care provided to patients. This information will support decisions on the focus of subsequent research, at the same time as providing efficient output for the network.

**Program approach:**

| ***Phase 1: Rapid review.***  *Aim:*   1. To identify and consolidate themes and domains for high value and high-quality care as defined in current literature. 2. Document network members' opinions about gaps in concepts of high value and high-quality care.   *Method*:   1. Rapid review to generate ‘RIPN statement on high value and high-quality care’. 2. Discussion on rapid review among RIPN SteerCo. 3. Network member’s opinions to be gathered with Google Forms and option to have online semi-structured interview.   *Outputs:*   - A draft document (consolidating common themes from definitions of high value care and high-quality care) - ***‘RIPN statement on high value and high-quality care’.*** - Physiotherapists opinions about what is missing from the consolidated model of high value and high-quality care and how the models apply to patients with musculoskeletal pain. - An infographic outlining ‘definitions’ and ‘common themes. - A video abstract from rapid review - A published report or scientific paper on the process and rapid review results |
| --- |

| ***Phase 2: Formal scoping review***  Aim:   1. Describe the type and details of evidence published on the use or implementation of high value and high-quality care for musculoskeletal conditions. 2. Assess alignment and describe differences between models of high value and high-quality care used for implementation within in MSK research and the RIPN proposed model.   *Method*: Scoping review  *Output:*   - Published scoping review. - Other dissemination of results |
| --- |

| ***Phase 3: Mixed methods work to explore barriers, enablers and strategies to implementing high value and high-quality care.***  *Aim:*  1) Explore barriers to implementing the definitions and themes identified from phase 1  2) Brainstorm strategies to overcome these barriers  Aims for patients:   1. Explore patients' thoughts, feelings, preferences in high value and high-quality care (How do patients define these concepts? What do these terms mean to them?) 2. Explore the differences, discrepancies in these preferences (above) and ‘RIPN consensus definitions and themes’ of high value and high-quality care.   *Method:* Online survey and online focus groups with the following 5 sample groups:   1. Patients 2. Other physiotherapists (not in the network) 3. Other primary care clinicians (GPs) 4. Tertiary level clinicians (Surgeons, EPs)   **Option: Industry stakeholders (rehab providers, private health insurance representatives)  *Output/s:*   - A list of barriers to implementing high value and high-quality care - A list of co-designed strategies to assist in implementing high value and high-quality care (which map to barriers) - Any changes necessary to ‘RIPN statement on high value and high-quality care document’ - Publishable report - Implementation actions (form the basis of implementation trials) - Other dissemination of phase 3 |
| --- |

| ***Phase 4: Design of implementation trial based on phases 1-3.*** |
| --- |
