# Supplementary material for: Meaningful coproduction with clinicians: establishing a practice-based research network with physiotherapists in regional Australia
Source: Health Res Policy Syst. 2023 May 26;21:38. doi: 10.1186/s12961-023-00983-x (PMC10223842; doi:10.1186/s12961-023-00983-x)
Supplement: Supplementary file 3 — Additional file 3. Overview of the Network's initialresearch program. [file 12961_2023_983_MOESM3_ESM.docx]

**Additional file 1:** Focus group information sheet and interview schedule

**Information sheet for ‘Improving the quality of physiotherapy research and care for musculoskeletal pain across Hunter New England’: Focus groups**

Too often research does not address clinically important problems. This is because clinicians do not have a voice in what research should be undertaken. New South Wales (NSW) Regional Health Partners (RHP) have approached the Hunter New England Population Health (HNEPH) Musculoskeletal Team to help establish a network of physiotherapists across Hunter New England and support them to become engaged users and producers of practice-relevant research. The focus of this project is to better understand the challenges, barriers, and enablers that physiotherapists experience in engaging with research, and identify research priorities that are practice relevant.

**Who is running the project?**

Hunter New England Population Health (HNEPH) and NSW RHP.

**Who will be asked to participate?**

Please take part in this project if you:

- Are a registered physiotherapist working within the Hunter New England region.

**What does participation involve?**

Participants will be asked to take part in a short online focus group (no longer than 30 mins) to comment on their views and ideas of how physiotherapy research should proceed in the region. There will be no more than 6 other people in the online focus group with you and you will be given plenty of time to discuss issues important to you. It will be led by Hunter New England Population Health staff with experience in this area.

**What will happen to the information you give us?**

All the answers provide to the project team will be treated in STRICT CONFIDENCE. The focus groups will be recorded so that they can be transcribed at a later date. We will then transcribe this video and audio file into a word document and immediately delete this recording. You can turn off your camera at any time you wish, or simply partake in the focus group through your computer audio. The only information collected about participants will be region of practice and experience level (in years). No identifying information (e.g. names) will be collected about participants. Reports that are based on the responses will not identify individuals, but may quote de-identified responses.

**Do you have to talk to us?**

It is entirely your choice whether you participate in this project. You do not have to answer our questions if you do not want to. **If you decide to not participate, it will NOT affect your relationship with Hunter New England Local Health District, Hunter New England Population Health or New South Wales Regional Health Partners.**

**What’s next?**

We will next send out an online survey asking for physiotherapist’s research priorities through the same social media groups, in which this focus group was advertised. You do not have to take part in this online survey if you do not wish.

We look forward to working with you to improve the relevance and quality of physiotherapy research, and improve the quality of care for musculoskeletal pain. Please do not hesitate to contact Dr Christopher Williams if you have any questions or concerns.

Yours Sincerely

Dr Christopher Williams

Hunter New England Population Health

Ph. (02) 4924 6121

Fax. (02) 4924 6048

**Project Team**

A/Prof Chris Williams Connor Gleadhill Professor Christine Jorm

Program Manager Physiotherapist/ PhD Candidate Director

HNE Population Health HNE Population Health NSW Regional Health Partners

University of Newcastle University of Newcastle University of Newcastle

Prof Steven Kamper Dr Hopin Lee

Clinical Research Fellow Research Fellow

Institute for Musculoskeletal Health University of Oxford

University of Sydney University of Newcastle

This research has been approved by the Hunter New England Human Research Ethics
Committee of Hunter New England Local Health District, Reference 2020/ETH01029
 
Should you have concerns about your rights as a participant in this research, or you have a
complaint about the manner in which the research is conducted, it may be given to the
researcher, or, if an independent person is preferred, to Dr Nicole Gerrand, Manager
Research Ethics and Governance, Hunter New England Local Health District,
HMRI Building Level 3 POD HMRI Lot 1 Kookaburra Circuit 
New Lambton NSW 2305, telephone (02) 49214950, email HNELHED-HREC@health.nsw.gov.au

Interview schedule

| Section I. How do we improve practice  We understand a lot of our patients have a rough ride through the healthcare system and there are many factors involved in providing ‘high value’ care.   1. **How do we improve practice?**   Section II. Opinion on research  **2) Where does research fit into this?**  Probe (if needed) - What is the purpose of research?  Section III. Problems  **3) What type of issues do you think this network could help with?**  Section IV. Clinician’s solutions  **4) In your eyes, how do you see this network being successful?**  Probes - What would you need? Time? Resources? Face to face/online?  Section V. **‘other’ problems** and whether network is the right place to address them  Let’s step away for a while from the idea of the practice-based network. In our earlier discussions with physios, we’ve heard about problems that clinicians face related to running their business, or those related to communication issues with other professionals, e.g. doctors. Non-clinical problems.  **5) Is that something that you experience as well?**  6) (if yes) Can you think of other ways to address these problems? (They don’t have to involve a PBRN)  (if no- don’t go any further)  Would practice-based network be helpful in addressing these problems at all? If yes, why/how? If no, why not? What else would help you address these problems? |
| --- |
